# Receptor Tyrosine Kinase EphA5 Is a Functional Molecular Target in Human Lung Cancer*

**DOI:** 10.1074/jbc.M114.630525

**Published:** 2015-01-26

**Authors:** Fernanda I. Staquicini, Ming D. Qian, Ahmad Salameh, Andrey S. Dobroff, Julianna K. Edwards, Daniel F. Cimino, Benjamin J. Moeller, Patrick Kelly, Maria I. Nunez, Ximing Tang, Diane D. Liu, J. Jack Lee, Waun Ki Hong, Fortunato Ferrara, Andrew R. M. Bradbury, Roy R. Lobb, Martin J. Edelman, Richard L. Sidman, Ignacio I. Wistuba, Wadih Arap, Renata Pasqualini

**Affiliations:** From the a University of New Mexico Cancer Center and; the Divisions of b Molecular Medicine and; l Hematology/Medical Oncology, Department of Internal Medicine University of New Mexico School of Medicine, Albuquerque, New Mexico 87131-0001,; the Departments of c Genitourinary Medical Oncology,; d Radiation Oncology,; e Translational Molecular Pathology,; f Biostatistics, and; g Thoracic/Head & Neck Medical Oncology, David H. Koch Center, University of Texas M. D. Anderson Cancer Center, Houston, Texas 77030,; the h Bioscience Division, Los Alamos National Laboratory, Los Alamos, New Mexico 87545,; i Alvos Therapeutics, Arrowhead Research Corporation, Pasadena, California 91101,; the j University of Maryland School of Medicine, Baltimore, Maryland 21201, and; the k Department of Neurology, Beth Israel Deaconess Medical Center, Harvard Medical School, Boston, Massachusetts 02215

**Keywords:** Cell Cycle, DNA Damage, DNA Damage Response, DNA Repair, Monoclonal Antibody, Receptor Tyrosine Kinase, Ionizing Radiation

## Abstract

Lung cancer is often refractory to radiotherapy, but molecular mechanisms of tumor resistance remain poorly defined. Here we show that the receptor tyrosine kinase EphA5 is specifically overexpressed in lung cancer and is involved in regulating cellular responses to genotoxic insult. In the absence of EphA5, lung cancer cells displayed a defective G_1_/S cell cycle checkpoint, were unable to resolve DNA damage, and became radiosensitive. Upon irradiation, EphA5 was transported into the nucleus where it interacted with activated ATM (ataxia-telangiectasia mutated) at sites of DNA repair. Finally, we demonstrate that a new monoclonal antibody against human EphA5 sensitized lung cancer cells and human lung cancer xenografts to radiotherapy and significantly prolonged survival, thus suggesting the likelihood of translational applications.

## Teaser

**Background:** EphA5 is a functional target in lung cancer, the most common cause of tumor-related death in mankind.

**Results:** EphA5 regulates cell cycle checkpoints and DNA damage repair induced by ionizing radiation.

**Conclusion:** EphA5 is a novel regulator of DNA damage repair with clinical implications.

**Significance:** EphA5 may serve as a novel biomarker of radioresistance and a candidate target for therapeutic intervention in human lung cancer.

## Introduction

The concept of targeted cancer therapy is predicated on the assumption that tumors have unique and sustained genetic abnormalities and that direct targeting of such distinctive biological features can only be accomplished through the identification and validation of certain specific molecular markers. These principles are particularly relevant to lung cancer, the leading cause of cancer-related death worldwide (1–3). For patients with early stage disease, surgical resection or primary radiotherapy are generally the standard treatments, whereas combined modality therapy (radiation plus chemotherapy, with or without surgery) is preferred for locally advanced disease (4, 5). However, most patients with lung cancer develop metastatic tumors and resistance to therapy and ultimately die of their disease.

Upon genotoxic stress such as ionizing radiation (IR),^6^ cells use molecular signaling networks to sense, interpret, and respond to breaks in DNA, whether single-stranded or double-stranded. Following the detection of DNA lesions, the DNA damage response (DDR) coordinates DNA repair, cell cycle checkpoints, and specialized programs such as apoptosis and senescence (6). Such complex signaling cascades have traditionally been divided into two major protein kinase pathways: one primarily mediated by the ATM (ataxia-telangiectasia mutated) kinase protein and the other primarily mediated by ATR (ataxia-telangiectasia and rad-3-related) kinase (7–9). Once activated, ATM phosphorylates several downstream substrates, including p53 and Mdm2, molecular modifications that lead to IR-induced arrest of cells in G_1_, S, or G_2_ cell cycle phases (10–13). Cells that are functionally defective in components of the DDR pathways show cell cycle checkpoint defects, a decreased capacity to repair DNA, and an increased sensitivity to IR and other DNA damaging agents (6). Hence, new tools that impair the response of cancer cells to DNA damage could advance the development of anti-cancer therapies.

We searched for new molecular targets specific for human tumors by high throughput combinatorial screening of a phage-displayed random peptide library to classify human tumor cell lines according to the binding selectivity of recovered peptide motifs (14). One group of tumor cell-binding peptides exhibited targeting specificity for human lung cancer cells. Identification of some of these binding peptides led to the suggestion that the corresponding candidate surface receptor was likely the RTK EphA5 (14). Ephrin receptors belong to a family of closely related proteins with diverse functions in both normal physiology and disease pathogenesis (15, 16). EphA5 is mostly recognized for its critical role in axonal guidance during embryonic development (17–19); its involvement in cancer is only now becoming evident (20–22).

Here, we identify EphA5 as a molecular target of lung cancer and as a novel regulator of IR-induced cell cycle checkpoint and DNA damage repair, with unexpected roles in the resistance of lung cancer to radiotherapy. In addition, we produced a new monoclonal antibody against EphA5 that sensitizes lung cancer cells to IR *in vitro* and improves the overall survival of mice bearing human lung cancer xenografts in combination with radiation therapy. These findings present a readily available and potentially effective strategy for the detection and treatment of lung cancer.

## EXPERIMENTAL PROCEDURES

### 

#### 

##### Animals

NU/NU nude mice and Balb/c mice (Harlan Laboratories) were housed in the animal facility of the University of Texas M. D. Anderson Cancer Center (Houston, TX). All animal procedures were reviewed and approved by the Institutional Animal Care and Use Committee of the M. D. Anderson Cancer Center. Nude rats were also purchased from Harlan Laboratories and housed in the animal facility of MPI Research. The imaging studies in nude rats were conducted at MPI Research (Boston, MA) and were reviewed and approved by the Institutional Animal Care and Use Committee. MPI Research is accredited by the Association for Assessment and Accreditation of Laboratory Animal Care International. The facility maintains an Animal Welfare Assurance statement with the National Institutes of Health Office of Laboratory Animal Welfare. To ensure compliance, the protocol was reviewed and approved by the Institutional Animal Care and Use Committee before any investigational work was conducted.

##### Reagents

The following reagents were used: rabbit polyclonal anti-EphA5 antibody (Santa Cruz Biotechnology, clone L15), rabbit monoclonal anti-ATM IgG (Millipore, clone Y170), rabbit polyclonal anti-ATM (Millipore), mouse monoclonal anti-ATM IgG (Abcam), mouse monoclonal anti-phospho-ATM IgG (Ser^1981^) (Millipore, clone 10H11.E12), rabbit monoclonal anti-phospho-Chk2 IgG (Cell Signaling), rabbit monoclonal anti-Chk2 IgG (Cell Signaling), rabbit polyclonal anti-phosphotyrosine HRP-conjugated antibodies (Santa Cruz Biotechnology), and goat polyclonal anti-GST antibodies (GE Healthcare Life Sciences). Goat anti-rabbit HRP-conjugated, rabbit anti-goat HRP-conjugated, and rabbit anti-mouse HRP-conjugated IgGs, and ChromPure mouse IgG were purchased from Jackson ImmunoResearch Laboratories. Alexa Fluor® 488 rabbit anti-mouse IgG was purchased from Invitrogen. Cy-3- and FITC-conjugated anti-mouse and rabbit IgG were purchased from Jackson ImmunoResearch Laboratories. Recombinant human EphA5 extracellular domain was purchased from R&D Systems. The complete EphA5 cytoplasmic domain (Cellsciences) and EphA5 kinase domain (Millipore) were also obtained commercially.

##### Cell Culture

293GPG cells were cultured in high glucose DMEM containing 10% heat-inactivated FBS and 1 μg/ml tetracycline (Sigma) at 37 °C and 5% CO_2_. NCI-H460, H1299, A549, NCI-H226, NCI-H23, and NCI-H522 human lung cancer cell lines were purchased from the American Type Culture Collection and maintained in DMEM containing 10% FBS and antibiotics. After lentiviral infection, stably transfected cells were selected and maintained in medium containing puromycin (Sigma) or puromycin plus blasticidin (Sigma). Normal human pulmonary fibroblasts were obtained from PromoCell and were cultured in supplemented fibroblast growth medium (PromoCell). Hybridomas were kept in RPMI 1640 medium supplemented with 10% FBS and antibiotics.

##### Lentiviral shRNA and Expression Vector Construct

Human EPHA5 targeting shRNA (TRCN0000006413, referred to as EphA5-shRNA1 and TRCN0000006415, referred to as EphA5-shRNA2) cloned into the retroviral pLKO.1 vector were purchased from the TRC lentiviral shRNA library (Open Biosystems). Ready to use lentiviral particles expressing a validated p53-shRNA (LVP343-GB) or the human gene TP53 (LVP253, NM_000546), and lentiviral controls (LVP-Null-RB and LVP-Ctr-GB) were purchased from AMSbio (Lake Forest, CA). EPHA5-shRNA lentiviruses were produced by transient transfection of human embryonic kidney cells (293FT; Invitrogen) with the sequence-verified pLKO.1 vector containing the EPHA5-shRNA sequence. Forty-eight hours later, the viral supernatant was collected, filtered to remove cellular debris, and aliquoted. Cells plated at 70% confluence in 6-well plates were infected with lentiviruses, and after 16 h, the virus-containing medium was removed and replaced with normal growth medium. Stably transduced cells were selected by addition of either puromycin (2 μg/ml) or blasticidin (60 μg/ml) to the growth medium. Rescue of EphA5 expression in EphA5-silenced cells was performed with an expression vector containing the human EphA5 receptor cDNA ORF (NM_004439.6), purchased from Origene (RG213206). The EphA5 cDNA was digested with AsiSI (NEB, R0630S) and MluI (NEB, R0198S) restriction enzymes and subcloned into a compatible pLenti-C-TurboGFP expression vector system (Origene, PS100065). Lentiviral particles containing the EphA5 cDNA were prepared following the protocol described previously. H460 EphA5-shRNA or NT-shRNA were plated in 6-well plates and transduced with the virus containing either the EphA5 expression vector or empty vector. Transduced cells were sorted based on expression of GFP. EphA5 expression was confirmed by immunoblotting.

##### Protein Extracts, Immunoblotting, and Immunoprecipitation

Extraction of total protein was performed with Nonidet P-40 extraction buffer containing protease inhibitors (50 mm Tris buffer, pH 8.0, 150 mm NaCl, and 1% Nonidet P-40). For immunoprecipitation assays, cells were lysed in Nonidet P-40 extraction buffer and, after overnight incubation with antibody-loaded magnetic beads (2 mg of total protein/1 μg of antibody), washed extensively in wash buffer (50 mm Tris buffer, pH 8.0, 0.5 m NaCl, and 1% Nonidet P-40). For protein detection by immunoblotting, proteins were resolved on 4–12% NuPAGE® Novex Bris-Tris gels (Invitrogen), transferred to nitrocellulose membranes (Bio-Rad), blocked with PBS containing 5% nonfat milk, incubated with primary antibody, washed, incubated with HRP-conjugated secondary antibody, and developed with an enhanced chemiluminescence reagent.

##### Subcellular Fractionation

Fractionation of DNA-associated proteins was performed as follows: nonirradiated control cells or cells treated with IR were washed once with ice-cold PBS and resuspended in extraction buffer (10 mm HEPES, pH 7.9, 10 mm KCl, 1.5 mm MgCl_2_, 340 mm sucrose, 10% glycerol, 1 mm DTT) containing protease and phosphatase inhibitors. After a short incubation period, nuclei were collected by centrifugation at 1,300 × *g* for 5 min and washed once with extraction buffer. Nuclear proteins were resuspended in a second extraction buffer (3 mm EDTA, 0.2 mm EGTA, 1 mm DTT) containing protease and phosphatase inhibitors and were incubated at 4 °C for 30 min. Soluble and insoluble fractions were obtained by centrifugation at 1,700 rpm for 5 min at 4 °C. Finally, the pellet containing insoluble proteins was resuspended in extraction buffer and sonicated.

##### Real Time Quantitative PCR Analysis

Two sets of total RNA were independently isolated from cells with the RNeasy mini kit (Qiagen). For DNA synthesis, 500 ng of RNA from each sample were denatured at 70 °C for 5 min and quickly chilled on ice. First strand cDNA synthesis was carried out for 2 h at 42 °C with the ImProm-II^TM^ reverse transcription system (Promega). Quantitative real time PCR analysis was performed in a 7500 Fast Real-Time PCR System instrument (Applied Biosystems). Probes were as follows: Hs00178313_m1 for EPHA1, Hs00171656_m1 for EPHA2, Hs00739096_m1 for EPHA3, Hs00177874_m1 for EPHA4, Hs00300724_m1 for EPHA5, Hs00297133_m1 for EPHA6, Hs00177891_m1 for EPHA7, Hs01025610_m1 for EPHA8, Hs00543478_m1 for EPHA10, Hs00358886_m1 for EFNA1, Hs01023290_m1 for EFNA2, Hs00191913_m1 for EFNA3, Hs00193299_m1 for EFNA4, Hs00157342_m1 for EFNA5, Hs00270004_m1 for EFNB1, Hs00970625_m1 for EFNB2, Hs00154861_m1 for EFNB3, Hs01057849_m1 for EPHB1, Hs00362096_m1 for EPHB2, Hs00177903_m1 for EPHB3, Hs00174752_m1 for EPHB4, and Hs01071144_m1 for EPHB6. The gene expression ratio was normalized to that of 18 S.

##### Colony Formation in Soft Agarose

Cells were suspended at the concentration of 5000 cells/2 ml of 0.35% low melting agarose in a 35-mm culture dish containing 0.7% agarose base. Triplicates were prepared for each tested cell line and inspected by eye after staining with crystal violet for colony formation after incubation at 37 °C for 14–21 days.

##### ELISA-based Protein Interaction and Solution Binding Assay

Immunocapture of ATM and pATM was performed with Reacti-bind protein A plates (Pierce) coated with anti-pATM, anti-ATM, or IgG control antibodies, as described (23). ELISA with anti-IgG confirmed equal molar concentrations of IgG in each of the wells. After a blocking step with PBS containing 3% BSA, 30 μg of protein from total cell extracts was added to the wells for overnight incubation at 4 °C. After gentle washes, tagged EphA5 constructs were added to each well; after overnight incubation and several washes in PBS, the assay was developed with HRP-conjugated secondary antibodies against the tags. Solution binding assays were performed in binding buffer (20 mm HEPES, pH 6.8, 150 mm KOAc, 2 mm Mg(OAc)_2_, 2 mm DTT, 0.1% Tween 20). For each experiment, EphA5 constructs were incubated with anti-pATM or anti-ATM antibodies or control IgG-coupled magnetic beads in 500 μl of binding buffer overnight at 4 °C. At the end of the incubation period, beads were collected by centrifugation, and unbound proteins in the supernatant were collected by removal of 28 μl from the meniscus (corresponding to the unbound fraction). After two washes with 500 μl of binding buffer, the beads were resuspended in 20 μl of buffer. All samples were processed by the addition of sample buffer containing β-mercaptoethanol and by heating at 95 °C for 5 min. Proteins were resolved on NuPAGE® gels, transferred to nitrocellulose membranes, and analyzed by immunoblotting.

##### Clonogenic Assay

Cells were removed and plated sparsely on 6-well plates (Falcon) (250 or 500 cells/well) or 100 × 15mm Petri dishes (BD Falcon) (5,000 cells/dish) and were exposed to the specified doses of IR. Ten days later, cells were fixed and stained in 50% methanol in water containing 0.5% crystal violet to facilitate counting of colonies (≥50 cells). Clonogenic survival was calculated for each IR dose after normalizing for plating efficiency. The surviving fraction is the plating efficiency of treated cells divided by the plating efficiency of controls multiplied by 100.

##### ATM Foci Formation

Cells were plated in 16-well chambers (Lab-Tek®) at 10,000 cell/well. After overnight incubation, cells were treated with 3 Gy of IR and fixed in methanol at different time points after irradiation. Cells were blocked for 2 h with 1% BSA in PBS, followed by overnight incubation with primary antibodies diluted in 1% BSA in PBS. After three washes with PBS, cells were incubated for 30 min with FITC-conjugated anti-rabbit and Cy3-conjugated anti-mouse antibodies and diluted in 1% BSA in PBS. Cells were washed three times with PBS and were mounted with mounting reagent containing DAPI (Vectashield, Vector Labs). Cells were viewed under a 60× oil immersion objective. Images were recorded, pseudo-colored, and merged. For quantification, ATM foci in the nuclei of at least 50 cells (random fields) were counted under the microscope for each experimental condition.

##### Cell Cycle Analysis

Cells were partially synchronized in 0.2% BSA in DMEM and were stimulated with 10% FBS 24 h prior to IR treatment. For evaluation of DNA content, cells were treated, removed, and fixed in 70% ice-cold ethanol during vortexing. Approximately 30 min before analysis, cells were incubated in a solution of PBS containing 1% BSA and 50 μm propidium iodide. Samples were analyzed on a FACSCanto II instrument (BD Biosciences). Cell cycle analysis was performed using the FlowJo DNA/Cell Cycle platform.

##### SA-β-gal Staining

Staining of subconfluent cultured cells with a β-gal staining kit (Cell Signaling) was performed 7 days after treatment with IR (5 Gy). Percentages of SA-β-gal-positive cells were determined by scoring 3,000 cells (three independent fields of 1,000 cell each), in two independent experiments. For evaluation of senescence *in vivo*, tumor samples were collected 10 days after the end of IR treatments. Tissue samples were snap frozen in liquid nitrogen, embedded in OCT, and sectioned immediately. Tissue sections were stained with the same β-gal staining kit and counterstained with eosin. Percentages of SA-β-gal-positive cells were determined by scoring 1,000 cells in triplicate (three independent fields, for a total of 3,000 cells scored). Five tumors were evaluated for each experimental group.

##### Monoclonal Antibody Production

Balb/c mice were immunized three times at 14-day intervals with 12 μg of recombinant extracellular EphA5 dissolved in complete Freund's adjuvant. Sera collected from tail veins were tested by ELISA after the third immunization. Splenocytes of mice with high antibody titers were fused with mouse myeloma cells (P3X63Ag8.653, American Type Culture Collection) (4:1 ratio) with polyethylene glycol (molecular weight = 1500; Sigma). After fusion, cells were cultured in 96-well plates at 1 × 10^5^ cells/well in RPMI 1640 selection medium containing 20% FBS, 10% hybridoma supplements (Sigma), 2 mm l-glutamine, 100 units/ml penicillin G, 100 mg/ml streptomycin SO_4_, 10 mm HEPES, and hypoxanthine-aminopterin-thymidine (Sigma). Selected hybridomas were subcloned four times by limiting dilution. Conditional media were removed from each stable hybridoma culture. The Ig class of mAb was determined with a mouse mAb isotyping kit (Santa Cruz). ELISA, flow cytometry, and immunoprecipitation were used to characterize the monoclonal antibody 11C12. The epitope recognized by 11C12 was determined by peptide mapping and ELISA. In brief, GST-tagged peptides covering residues 304–467 of the extracellular portion of human EphA5 were produced and purified using a GST-tagged protein purification kit (GE Healthcare Life Sciences). Purified GST peptides were coated on a 96-well plate and blocked with BSA followed by addition of decreasing concentrations of 11C12. Recombinant human EphA5 was used as positive control. Binding to GST alone was used to adjust for nonspecific interactions.

##### Receptor-mediated Internalization and Degradation

Antibody internalization was evaluated by confocal microscopy. In brief, cells were placed in eight-well confocal chamber slides and were exposed to various concentrations of 11C12 or controls at 4 °C to allow for binding to the cell surface. After 30 min, cells were washed extensively and transferred to 37 °C for internalization. Cells were fixed, permeabilized, and stained with the appropriate secondary reagents. Receptor degradation was evaluated by immunoblotting. Cells were treated with 1 μg/ml of 11C12 or control IgG and collected after 30 min and 1, 3, 6, and 24 h of treatment. Nontreated cells were also used as control.

##### Immunohistochemistry and High Density Tissue Microarray (TMA)

EphA5 immunostaining was performed on an autostainer (Dako Corporation). Human non-small cell lung cancer TMA specimens were from the Lung Cancer Specialized Program of Research Excellence Tissue Bank at the M. D. Anderson Cancer Center (24). Prior to the construction of the TMA, tumor tissue specimens of 198 lung cancers (125 ACC and 73 SCC) were examined histologically and were classified according to the WHO system. Detailed clinical and pathologic information, including demographic data, smoking history, pathologic tumor node metastasis staging, overall survival, and time of recurrence, was available in most cases. Hematoxylin was used for counterstaining. Immunohistochemical expression of EphA5 was quantified by two expert lung cancer pathologists (I. I. W. and M. I. N.) according to a four-value intensity score (0 for negative, 1 for weak, 2 for moderate, and 3 for strong), and the percentage of tumor cells within each category was independently estimated (0–100%). A final score was obtained by multiplying both intensity and extension values (0 × % negative tumor cells + 1 × % weakly stained tumor cells + 2 × % moderately stained tumor cells + 3 × % strongly stained tumor cells). Thus, the score ranged from a minimum of 0 to a maximum of 300.

Evaluation of EphA5 expression in a second cohort of patients that had previously been treated with radiotherapy was also performed. All patient information and materials were collected and handled in accordance with institutional review board-approved protocols and procedures. Patients with stage IIB-IIIB non-small cell lung carcinoma treated with primary lobectomy or pneumonectomy followed by postoperative radiotherapy at the M. D. Anderson Cancer Center from 1998 to 2005 were considered for inclusion. Patients receiving radiation doses less than 50 Gy, with positive surgical margins or without surgical mediastinal lymph node evaluation, were excluded. After cross-referencing for archived tissue materials, there were 23 patients available for further analysis. Individual patient medical records were reviewed for pertinent demographic and treatment outcome data. Locoregional relapse was defined as biopsy-proven recurrence within the radiation field. After immunohistochemistry for EphA5 was performed, the cohort was divided into high *versus* low EphA5 expression on the basis of scores at or above *versus* below the median score (median = 130) for the group. Cumulative rates of locoregional relapse were plotted, and log rank analysis to detect differences between the groups was performed. Overall survival was estimated for the cohort as a function of EphA5 staining by Kaplan-Meier methodology, with the use of log rank analysis to detect differences between the groups (SPSS version 17).

##### In Vivo Tumor Irradiation

Gamma radiation was delivered locally to the tumors from a ^60^Co source, at the rate of 1 Gy/min with a custom head and body shielding. Tumor-bearing mice received fractionated radiation therapy of 3 Gy a day for 3 consecutive days, 6 h after treatment with either 11C12 or control IgG (5 mg/kg each). Control groups received vehicle alone or 11C12 alone. Tumor volumes were determined from digital caliper measurements and are reported as mean tumor volumes ± S.D.

##### Imaging Study of ^111^In-labeled Antibody in Nude Rats

Radiolabeling of 11C12 and control IgG was performed by inviCRO (Boston, MA). For DTPA conjugation, 30-kDa Amicon filter units were used to exchange 1 mg of IgG into a sodium bicarbonate buffer. This buffer exchange step was repeated five times. One mg of IgG was added directly to 500 μg of DTPA in a CryoVial tube and mixed by vortexing. The reaction was allowed to incubate at room temperature for 2 h. The reaction mixture was exchanged into 0.6 m ammonium acetate buffer with 30-kDa Amicon filter units. The product was rinsed five times with 0.6 m ammonium acetate buffer (pH 6.5) and was stored at 4 °C. For labeling of DTPA-IgG with ^111^In, 0.6 m ammonium acetate buffer was added to ^111^InCl_3_ until the solution reached pH 6.5. Approximately 5 mCi of ^111^In was added to a CryoVial tube containing 0.2 mg of DTPA-IgG, and the reaction mixture was incubated at 37 °C for 1.5 h. Following incubation, 0.1 m ammonium acetate buffer containing 50 mm EDTA was added to the reaction tube. The reaction mixture was exchanged into PBS with a 30-kDa Amicon filter unit. Radiochemical purity was confirmed by HPLC at ∼ 94%.

Six female nude rats were assigned to this study. Four nude rats were inoculated subcutaneously with H460 xenograft tumors; another two nude rats were used for biodistribution studies in non-tumor-bearing rats. Tumor-bearing rats were enrolled in the study when tumor volumes were 300–500 mm^3^. Nude rats were injected intravenously with ^111^In-DTPA-11C12 or ^111^In-DTPA-IgG (at a dose level of 0.6 mg/kg) and were imaged on a NanoSPECT/CT^TM^ (Bioscan) at 3, 24, 48, 96, and 144 h postinjection. Reconstructed images from the NanoSPECT/CT^TM^ were generated in units of activity (kBq or equivalent). Regions of interest were acquired in one of three manners: by fitting ellipsoids of fixed volume to the heart (surrogate end point), brain, liver, and kidneys; by fitting a sphere to a region defined as muscle; or by hand drawing, as in the case of the tumor region of interest (25).

##### Statistical Analysis

Survival probability as a function of time was computed by the Kaplan-Meier methodology. The log rank test was applied to compare survival curves among experimental cohorts of mice. EphA5 expression was compared between two groups by the Wilcoxon rank sum test or among three groups by the Kruskal-Wallis test. Association between EphA5 positivity (0 versus greater than 0) and other covariates was tested either by Fisher's exact test or chi-squared test. *p* < 0.05 was considered to be statistically significant. Statistical analyses were done with S-Plus and SPSS software.

## RESULTS

### 

#### 

##### EphA5 Is Expressed in Human Lung Cancer

**FIGURE 1.**
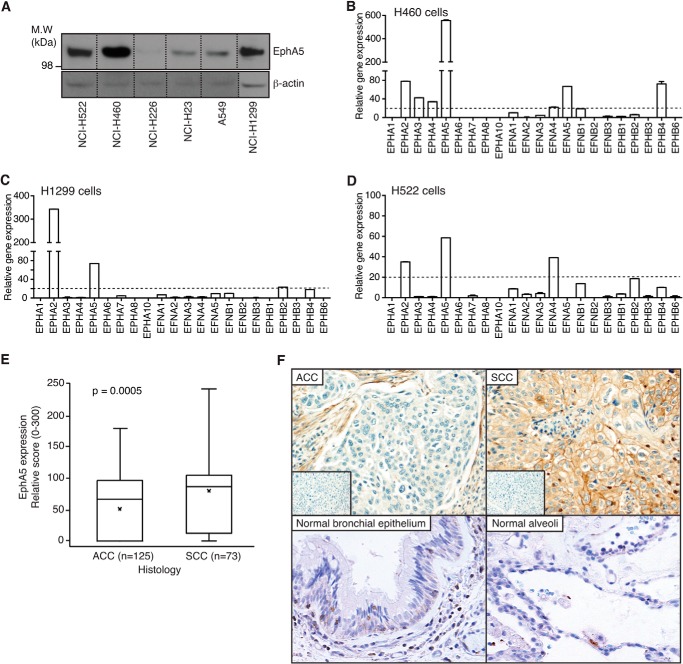
**EphA5 is expressed in human lung cancer cells and in patient-derived lung cancer specimens.** *A*, expression of EphA5 in a panel of human lung cancer cells. *B–D*, quantitative real time PCR of EPH receptors and ephrins in H460 (*B*), H1299 (*C*), and H522 (*D*) cells. *E*, overall assessment of EphA5 expression in ACC and SCC samples. High levels of EphA5 expression were predominantly associated with SCC *versus* ACC. *p* < 0.0005 (Wilcoxon rank sum test). Means are indicated by ×. *F*, representative microphotographs of EphA5 staining in ACC and SCC (magnification, ×200). *Insets*, irrelevant IgG used as negative control. *Bottom panels* show low expression of EphA5 in normal lung. *M.W.*, molecular mass.

To search for new molecular targets in human cancers, we have previously reported a high throughput combinatorial screening of a phage-displayed random peptide library to classify human tumor cell lines according to the binding selectivity of several thousands of recovered peptide motifs (14). One group of cell-binding peptides showed targeting specificity for human lung cancer cells; the corresponding surface receptor for some of these ligand peptides was suggested to be the RTK EphA5. Thus, to initiate the characterization of EphA5 as a potential molecular marker of lung cancer, we determined levels of EphA5 expression in several human lung cancer cell lines (Fig. 1*A*). NCI-H460, NCI-H1299, and NCI-H522 cells showed the highest levels of protein expression and were selected for validation studies. Gene expression analyses of members of the ephrin family confirmed high expression of EPHA5 mRNA in all three cell lines (Fig. 1, *B–D*).

Next, we analyzed EphA5 expression by immunohistochemical staining with a commercial antibody on a lung cancer TMA (Fig. 1, *E* and *F*). We ensured antibody specificity by testing the binding of the anti-EphA5 antibody in EphA5-silenced cells, in tissue sections of H460-derived xenografts, and in the presence of competing recombinant EphA5 (data not shown). The TMA comprised of a cohort of tumor specimens (*n* = 198) obtained from patients diagnosed with non-small cell lung carcinoma, including adenocarcinomas (ACC; *n* = 125) and squamous cell carcinomas (SCC; *n* = 73), of differing clinical stages (24). This large panel of patient-derived samples has well annotated clinical-pathologic features that include smoking history, recurrence, and survival data (24). The presence of EphA5 in these sets of samples was assessed according to scores of expression, and the data were independently quantified by two lung cancer pathologists (I. I. W. and M. I. N.).

Expression of EphA5 was analyzed using the median in each histology group. EphA5 was found to be expressed in human lung cancers (∼70%), and the intensity of staining was substantially greater in SCC (median = 86.67) compared with ACC (median = 66.67) (Wilcoxon rank sum test, *p* = 0.0005) (Fig. 1*E*). Photomicrographs in Fig. 1*F* illustrate the distribution of EphA5 in lung cancer and normal lung. In both ACC and SCC, EphA5 was present in the membrane and cytoplasm of cancer cells. In normal bronchial epithelium and alveoli, EphA5 was barely detectable.

##### EphA5 Regulates Radioresistance of Human Lung Cancer Cells

**FIGURE 2.**
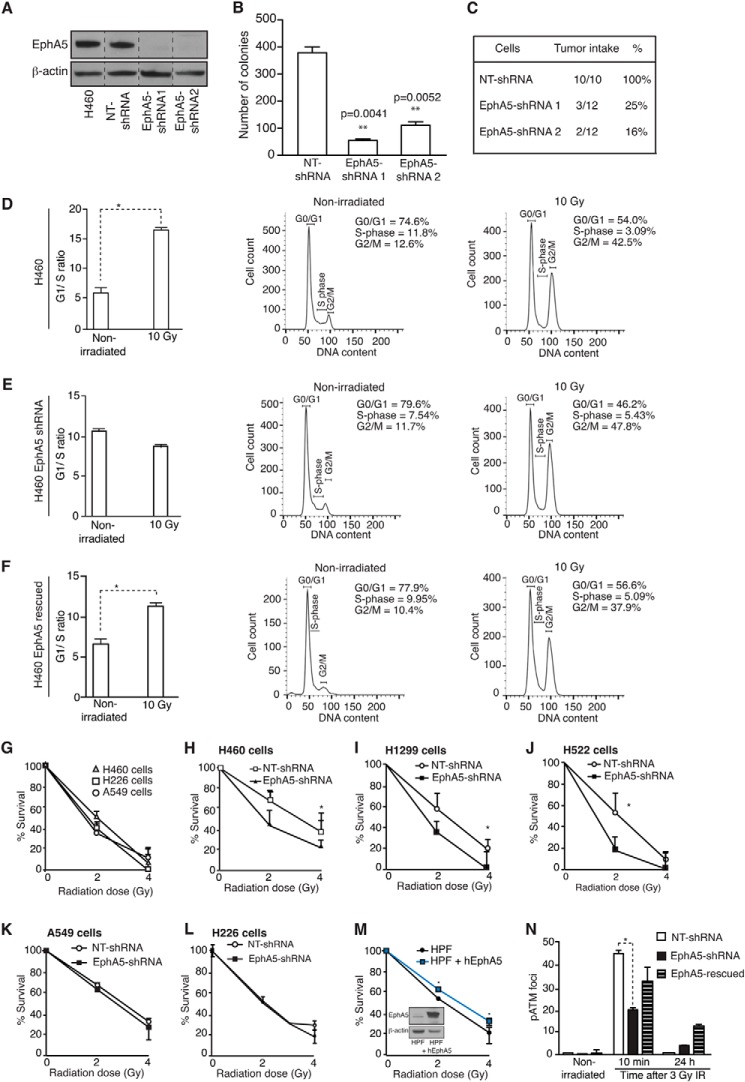
**Evaluation of EphA5 function in human lung cancer.** *A*, expression of EphA5 in H460 human lung cancer cells before and after shRNA silencing. *B* and *C*, soft agar assay (*B*) showing a reduced number of colonies formed after EphA5 silencing and tumor intake (*C*) after subcutaneous cell implantation in mice. *D–F*, cell cycle distribution of H460 lung cancer cells after IR. Fractions of H460 control cells (*D*), H460 EphA5-shRNA (*E*), and H460 EphA5-rescued cell (*F*) in G_1_ and S phases. EphA5-silenced cells do not arrest in G_1_ upon IR-induced DNA damage, as indicated by a 2-fold decrease in the G_1_ to S ratio. *, *p* < 0.05 (Student's *t* test). *G*, surviving fractions of A549 and H226 cells compared with H460 cells. *H–J*, surviving fractions of control and EphA5-shRNA cells as a function of increasing doses of IR. H460 (*H*), H1299 (*I*), and H522 (*J*). *Error bars* indicate S.D. Experiments were performed two times with similar results. *, *p* < 0.05. *K–M*, surviving fractions of A549 control and EphA5-shRNA cells (*K*), H226 control and EphA5-shRNA cells (*L*), and human pulmonary fibroblasts expressing or not human EphA5 (*M*) as a function of increasing doses of IR. *Error bars* indicate S.D. *N*, quantification of pATM foci in H460 control, EphA5-shRNA, and EphA5-rescued cells treated with 3 Gy of IR and evaluated 10 min and 24 h after irradiation. *Error bars* indicate S.D. Experiments were performed two times with similar results. *, *p* < 0.05.

Having confirmed expression of EphA5 in lung cancer cells and in a large cohort of patient-derived samples, we attempted to study the function of EphA5 in lung cancer. We initially tested two shRNA constructs targeting EphA5 (EphA5-shRNA1 and EphA5 shRNA2) in the human lung cancer cell line H460. Immunoblotting confirmed comparable reduction of EphA5 protein levels relative to the nontargeted control NT-shRNA and H460 wild-type cells (Fig. 2*A*). Shortly after silencing of EphA5, a delay in cell growth in culture was observed, suggesting potential alterations in cell division (data not shown). Concordantly, the ability of EphA5-silenced cells to grow colonies in soft agar and to form subcutaneous tumors *in vivo* was dramatically reduced (Fig. 2, *B* and *C*). Because of the similar phenotype observed with two EphA5-targeted shRNA sequences, all subsequent experiments were performed with EphA5-shRNA1 cells, hereafter denominated EphA5-shRNA.

These observations were followed by a functional evaluation of cell cycle checkpoint activity after IR-induced DNA damage in G_0_/G_1_-synchronized cells (Fig. 2, *D–F*). Exponentially growing cells were treated with IR to induce DNA damage and to activate the G_1_ checkpoint. The distribution of cells in the cell cycle phases (sub-G_1_, G_1_, S, and G_2_-M) was recorded, and the numbers of cells in G_1_ and S phases were used to calculate G_1_/S ratios to determine whether the G_1_-S cell cycle checkpoint was functional. Essentially, upon a genotoxic insult caused by IR, cells with an intact DDR will activate the G_1_ checkpoint to allow repair of DNA breaks. Consequently, a smaller fraction of cells will transition from G_1_ to S phase, thereby causing a higher G_1_/S ratio. If DDR is impaired, cells will not activate the G_1_ checkpoint, and as a consequence, a larger fraction of cells will transition from G_1_ to S phase despite the presence of DNA injury, thereby resulting in a lower G_1_/S ratio.

In comparison to nonirradiated cells, IR elicited cell cycle effects that were dependent on EphA5 expression. Control H460 cells were arrested in G_1_ after IR, a result suggestive of their capacity to sense DNA damage (Fig. 2*D*). In contrast, H460 EphA5-shRNA progressed through G_1_/S, a result indicative of critical defects in the assessment of DNA damage caused by IR (Fig. 2*E*). Phenotypic rescue with an exogenously expressed shRNA-insensitive human EphA5 (Fig. 2*F*) restored the ability of cells to respond to IR exposure, therefore ruling out off target effects. DNA histograms illustrate the distribution of cells across the cell cycle (Fig. 2, *D–F*). Together, we reasoned that these findings indicate that DNA damage-induced biochemical events triggering G_1_/S checkpoint in EphA5-negative cells might be defective.

To investigate further a potential role for EphA5 in the cellular response to DNA damage, we used a clonogenic (colony formation) assay to test the survival of cells expressing different levels of EphA5 upon IR treatment. Cells expressing low levels (A549) or lacking EphA5 expression (H226) were more sensitive to IR in comparison to H460 cells (Fig. 2*G*). Moreover, when compared with control NT-shRNA cells, H460 EphA5-shRNA cells showed a marked reduction in survival (Fig. 2*H*). Similar results were also obtained with two additional lung cancer cell lines: NCI-H1299 and NCI-H522 (Fig. 2, *I* and *J*). No differences in response to IR-induced DNA damage were detected when EphA5 was silenced in A549 (Fig. 2*K*) or H226 cells (Fig. 2*L*). Furthermore, exogenous expression of EphA5 in human pulmonary fibroblasts rendered the cells resistant to IR (Fig. 2*M*).

These findings were also validated by immunofluorescence to measure the formation of active DNA damage repair foci with an antibody against phosphorylated ATM (pATM). Quantification of active pATM foci 10 min after cell treatment showed a marked reduction in the number of nuclear foci in H460 EphA5-shRNA cells in comparison to control and EphA5-rescued cells (Fig. 2*N*). After 24 h, DNA damage was resolved in EphA5-shRNA cells, indicating that DDR in these cells is impaired but not completely abrogated. Together, these results establish EphA5 overexpression as a mechanism of cellular radioresistance, with potential implications for the therapeutic use of IR in lung cancer.

##### EphA5 Expression Negatively Correlates with Radiotherapy Response in Lung Cancer Patients

**FIGURE 3.**
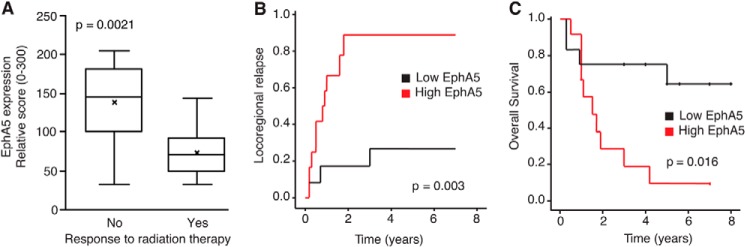
**Expression of EphA5 in patients receiving IR treatment prior to surgical resection of lung cancer.** *A*, high levels of EphA5 expression are directly associated with radiotherapy failure. *p* = 0.0021 (log rank test). *B* and *C*, locoregional recurrence (*B*) and cumulative overall patient survival (*C*) rates as a function of EphA5 expression.

Given the importance of EphA5 in resistance to IR treatment, we set out to validate these findings by examining the levels of EphA5 and its effect on response to radiation therapy in an independent cohort of surgically resected tumors from lung cancer patients (*n* = 23) with stage III non-small cell lung carcinoma, all of whom had undergone radiotherapy following “margin negative” surgery. The cohort included patients with biopsy-confirmed local failure within the radiation field (*n* = 12) and with no evidence of local failure (*n* = 11); histologies included ACC (74%) and SCC (26%), similarly distributed between the two groups (Fig. 3). EphA5 levels were significantly higher (*p* = 0.0021) in patients that failed radiation therapy, in comparison to those that responded well to the treatment (Fig. 3, *A* and *B*). More importantly, there was a direct correlation between EphA5 levels and mortality (Fig. 3*C*).

##### EphA5 Interacts with pATM in the Nucleus of Irradiated Lung Cancer Cells

**FIGURE 4.**
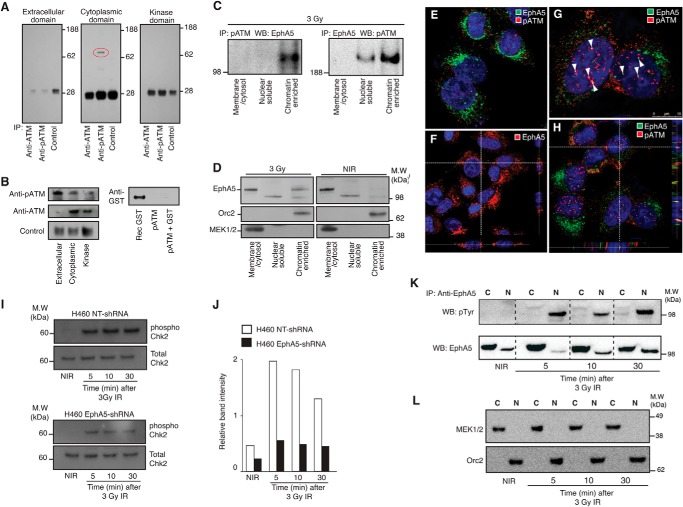
**EphA5 and ATM interact at sites of DNA damage repair.** *A* and *B*, a series of solution binding assays with pATM, ATM, and domains of EphA5 demonstrate that specific binding occurs only between pATM and the complete cytoplasmic domain of EphA5. *A*, *red oval* indicates the cytoplasmic domain of EphA5 coimmunoprecipitated (*IP*) with pATM. *B*, controls to confirm the presence of ATM and pATM in each experimental condition (*left panels*) and to rule out nonspecific interactions between pATM and the GST tag (*right panel*). *C*, immunoblot of fractionated irradiated cells immunoprecipitated with anti-pATM (*left panel*) or anti-EphA5 (*right panel*) antibodies. Coimmunoprecipitation of EphA5 and pATM is observed in both chromatin-enriched and nuclear soluble fractions. *D*, control nonirradiated H460 cells or cells treated with 3 Gy of IR were fractionated into membrane/cytosol, nuclear soluble, and DNA-associated proteins (chromatin-enriched). EphA5 expression is shown in the membrane/cytosol of control and treated cells. EphA5 translocation and interaction with DNA is observed only in IR-treated cells. Orc2 and MEK1/2 were used as controls for effective fractionation. *E–H*, confocal analysis of EphA5 expression and distribution in H460 cells before and after IR; DAPI staining of the nuclei is shown in *blue. E*, EphA5 and pATM in untreated cells. *F*, Z-stack of EphA5 expression in untreated H460 cells. *White dashed lines* point to orthogonal planes, showing EphA5 distribution in the perinuclear region of the cellular cytoplasm. *G*, coimmunostaining of EphA5 and pATM 10 min after cell irradiation. *White arrowheads* point to nuclear foci of active DNA repair containing colocalizing pATM and EphA5. *H*, orthogonal planes of a Z-stack image, confirming colocalization of EphA5 and pATM at nuclear sites of DNA damage repair. *Scale bar*, 10 μm. *I*, irradiation-induced phosphorylation of Chk2 is impaired in EphA5-silenced cells. Immunoblotting and quantification of protein bands show reduced phosphorylation of Chk2 in EphA5-shRNA cells compared with control cells (NT-shRNA). *K* and *L*, phosphorylation and nuclear import of EphA5. Cytoplasmic (*lanes C*) and nuclear (*lanes N*) proteins were isolated from nonirradiated H460 cells or from cells treated with IR and were subjected to immunoprecipitation with an anti-EphA5 antibody. Detection of phosphorylated and total EphA5 was performed with an anti-phosphotyrosine antibody or an antibody against EphA5 (*K*). Phosphorylated EphA5 was detected primarily in the nuclear fractions of irradiated H460 cells. The cytoplasmic protein MEK1/2 and the nuclear protein Orc2 (*L*) were used as control for fractionation efficiency.

Because our data implicated EphA5 in cell cycle control following IR and in intrinsic cellular radiosensitivity, we asked whether EphA5 and ATM would be molecular partners. We applied a solution binding assay using three constructs comprising the EphA5 extracellular domain (residues 25–573), the complete cytoplasmic domain (residues 595–1,037), and the kinase domain (residues 655–956) to determine whether ATM and EphA5 interact physically. These EphA5 domains were exposed to either pATM or ATM immobilized on magnetic beads. A clear interaction between pATM and the cytoplasmic domain of EphA5 was observed under these experimental conditions (Fig. 4, *A* and *B*). In contrast, no interactions were detected between EphA5 domains and nonphosphorylated ATM. Next, subcellular fractionation followed by coimmunoprecipitation was performed to demonstrate interaction of endogenous ATM and EphA5. Coimmunoprecipitation of EphA5 and pATM was detected in both nuclear soluble and chromatin-enriched protein fractions of irradiated cells, but not in the membrane/cytosol fraction (Fig. 4*C*).

Together, these results show a previously unrecognized interaction between EphA5 and phosphorylated ATM and also suggest the translocation of EphA5 to the nucleus upon IR. Indeed, cell fractionation studies confirmed the transport of EphA5 to the nucleus of irradiated cells and its association with chromatin (Fig. 4*D*, *left panel*). In contrast, the presence of EphA5 in nonirradiated cells was restricted to the membrane/cytosol fraction (Fig. 4*D*, *right panel*). The cytosolic protein MEK1/2 and the nuclear protein Orc2 were used as controls to assess purity. These findings were also confirmed by confocal microscopy. Prior to irradiation, pATM foci were at background levels (Fig. 4*E*), and EphA5 expression was detected mostly in or near the cell surface membrane, cytoplasm, and perinuclear regions, with background amounts of EphA5 observed inside the nucleus (Fig. 4, *E* and *F*). Ten minutes after cell treatment, ∼30% of nuclear repair foci contained colocalizing EphA5 and pATM (Fig. 4, *G* and *H*). Six hours later, EphA5 and pATM-containing nuclear foci were detectable, although in very small numbers (data not shown). Finally, and as yet more evidence of a functional relationship between EphA5 and ATM, we tested the ability of ATM to activate the downstream target Chk2, in the presence or absence of EphA5. In EphA5-silenced cells, phosphorylation of Chk2 is severely impaired after IR (Fig. 4, *I* and *J*).

Our findings thus far suggest that IR induces EphA5 nuclear localization, where it interacts with active ATM. Because standard ligand-dependent activation of the EphA receptors involves phosphorylation (7), we set out to determine the phosphorylation status of EphA5, specifically in cell nuclei. Nuclear extracts of irradiated and nonirradiated cells were immunoprecipitated with an anti-EphA5 antibody followed by immunoblotting with either anti-phosphotyrosine antibody or with a specific anti-EphA5 antibody. Upon irradiation, we observed an accumulation of phosphorylated EphA5 in the nucleus (Fig. 4, *K* and *L*). Again, the cytoplasmic protein MEK1/2 and nuclear protein Orc2 were used as control to fractionation efficiency.

##### EphA5 Acts through p53 to Control Cell Death and Cellular Senescence

The tumor suppressor protein p53 is a major downstream effector of ATM kinase pathways. In normal cells, p53-dependent signaling results in G_1_ arrest mediated mainly by transcriptional activation of p21. If the DNA damage is extensive, p53-dependent pathways target the damaged cell for apoptotic cell death through both the intrinsic and extrinsic pathways. Likewise, cancer cell growth can be inhibited by p53-mediated cell cycle arrest, apoptotic cell death, and/or cellular senescence.

**FIGURE 5.**
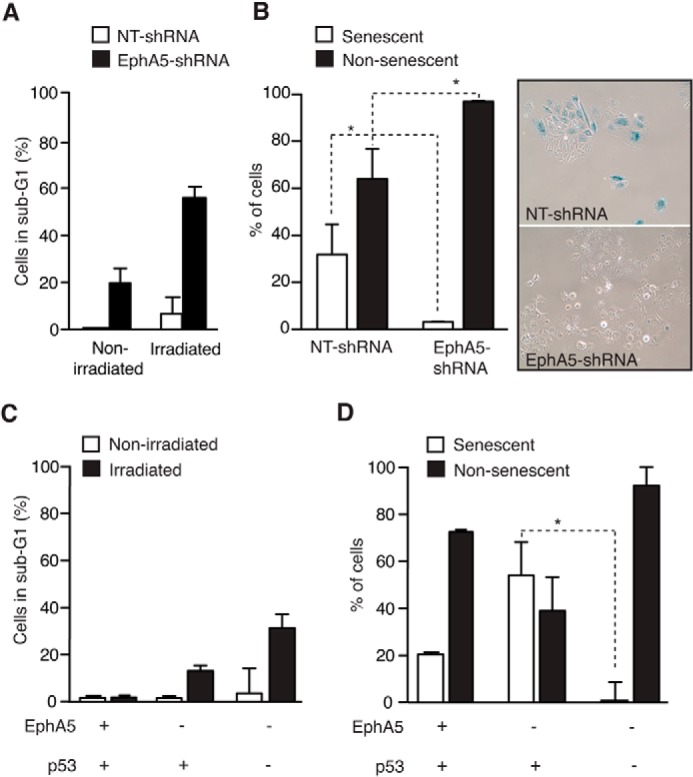
**EphA5 acts through p53 to control the fate of lung cancer cells.** *A*, percentage of apoptotic H1299 p53-null control and EphA5-shRNA cells before and after IR. Approximately 60% of H1299 EphA5-shRNA p53-null cells die after IR. *Error bars* indicate S.D. Experiments were performed three times with similar results. *B*, senescent H1299 control and EphA5-shRNA cells as determined by SA-β-gal staining. Panels show illustrative photomicrographs of cells stained for SA-β-gal activity postirradiation. *Error bars* indicate S.D. *, *p* < 0.05 (Student's *t* test). *C*, percentage of apoptotic H460 EphA5-shRNA p53-shRNA and control cells before and after IR. An increase of ∼30% in cell death is observed after IR. *Error bars* indicate S.D. Experiments were performed three times with similar results. *D*, the role of p53 in an EphA5-dependent cellular senescence pathway was studied in H460 EphA5-shRNA cells with or without p53-shRNA. The concomitant silencing of p53 and EphA5 abrogates the capacity of cells to become senescent. *Error bars* indicate S.D. *, *p* < 0.05 (Student's *t* test). Experiments were performed two times with similar results.

Thus, to ask whether p53 participates in EphA5/ATM-mediated functions, we used H1299 p53-deficient human lung cancer cells to study cell death and senescence. These cells were chosen because they express high levels of EphA5 (Fig. 1*A*) and because they carry a homozygous partial deletion of the p53 gene and therefore do not express the p53 protein. In H1299 EphA5-shRNA cells, the combined absence of p53 and EphA5 resulted in the death of ∼20% of cells, with a marked increase in cell death (60%) after exposure to IR (Fig. 5*A*).

We also asked whether senescence could be induced in the simultaneous absence of both EphA5 and p53. H1299 EphA5-shRNA p53-null cells and control cells were treated with IR, and cellular senescence was assessed by SA-β-gal activity. Notably, H1299 EphA5-shRNA p53-null cells did not become senescent (Fig. 5*B*). To confirm the role of p53 in the control of cell fate in the absence of EphA5, we used shRNA to silence p53 expression in H460 EphA5-shRNA cells. Coincident silencing of p53 and EphA5 resulted in a ∼30% increase in H460 cell death after IR (Fig. 5*C*). Moreover, H460 EphA5-shRNA p53-silenced cells did not become senescent after exposure to IR (Fig. 5*D*), results confirming our original observations.

Together, these findings support a novel and direct function of the RTK EphA5 in DNA damage response, with implications in the control of cell death and senescence. Based on our studies, the presence or absence of EphA5 dictates the terminal fate of IR-exposed lung cancer cells: in the absence of EphA5, p53-proficient cells become senescent, and in the absence of both EphA5 and p53, lung cancer cells die.

##### A New Monoclonal Anti-EphA5 Antibody Sensitizes Lung Cancer to IR

**FIGURE 6.**
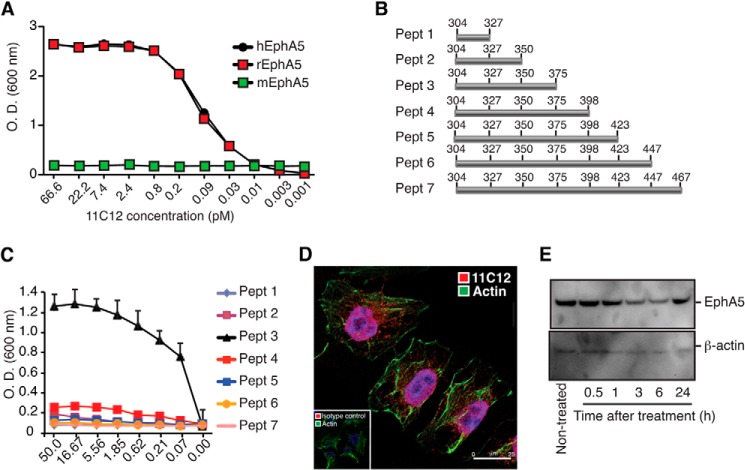
**Validation of anti-EphA5 monoclonal antibody 11C12.** *A*, ELISA showing binding of 11C12 to human and rat EphA5. The monoclonal antibody 11C12 does not recognize mouse EphA5. *B* and *C*, epitope identification was performed by peptide (*Pept*) mapping (*B*) using ELISA (*C*). *D* and *E*, receptor-mediated internalization (*D*) and receptor degradation (*E*) were also evaluated.

We hypothesized that inhibition of EphA5 activity would sensitize lung cancer cells to radiotherapy and offer a novel therapeutic strategy. Accordingly, we produced a panel of monoclonal antibodies against EphA5 and tested the binding specificity of each to endogenous EphA5. Hybridomas were prepared with splenocytes from a mouse immunized with the recombinant extracellular domain of EphA5 and screened by ELISA, flow cytometry, and immunoprecipitation (data not shown). The monoclonal antibody termed 11C12 was selected as the reference antibody for functional studies. This antibody recognizes both human and rat, but not mouse EphA5 (Fig. 6*A*).

We produced overlapping peptides covering residues 304–467 of the extracellular portion of human EphA5 (not present in mouse EphA5) and used ELISA to define the nature of the epitope bound by 11C12 (Fig. 6, *B* and *C*). The monoclonal antibody 11C12 reacted strongly with peptide 3, but not with any other peptide, a result suggestive of a largely conformational epitope contained within residues 304–375. In addition, we used confocal microscopy to evaluate receptor-mediated internalization (Fig. 6*D*), and immunoblotting to assess degradation of EphA5 upon 11C12 binding (Fig. 6*E*). Internalization of 11C12 was observed shortly after treatment (Fig. 6*D*). Antigen degradation is also detected at 3 and 6 h post-treatment (Fig. 6*E*).

**FIGURE 7.**
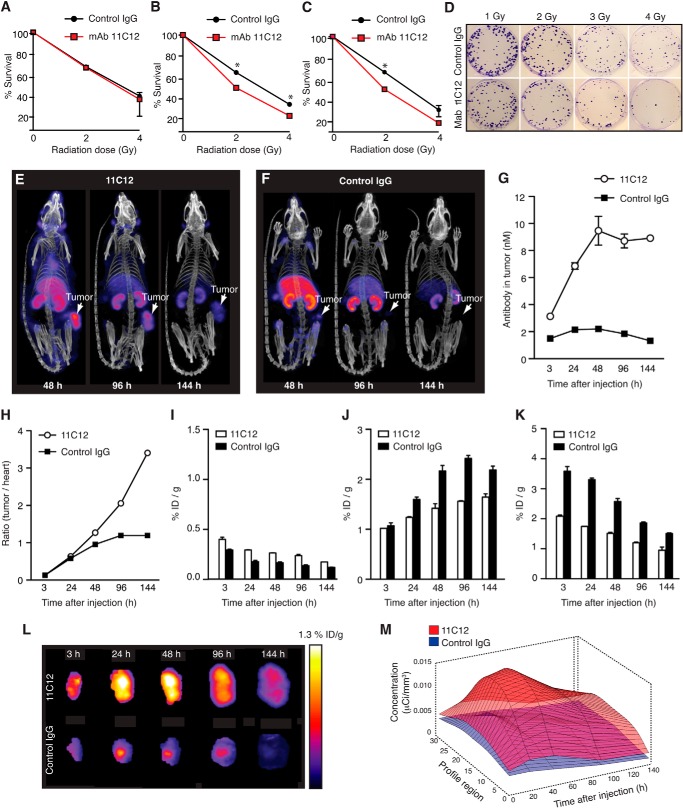
**The monoclonal antibody 11C12 radiosensitizes lung cancer cells *in vitro* and targets EphA5-expressing tumors *in vivo*.** *A*, surviving fraction of A549 cells (low EphA5 expression) treated with 11C12 or a control IgG. *B* and *C*, surviving fractions of H460 (*B*) and H522 (*C*) human lung cancer cells treated with the mAb 11C12 and subjected to increasing doses of IR. Isotype IgG was used as control. *, *p* < 0.05 (Student's *t* test). *D*, illustrative pictures of colony counts (H460 cells). Experiments were performed two times with similar results. *E* and *F*, illustrative SPECT/CT images of the distribution of ^111^In-DTPA-11C12 and ^111^In-DTPA-control IgG in tumor-bearing rats at 48, 96, and 144 h after antibody injection. *Arrows* point to the locations of the tumors. *G*, antibody concentration (nm) in the tumors as a function of time after injection. *H*, tumor to heart ratio of ^111^In-DTPA-11C12 and ^111^In-DTPA-control IgG as an indication of targeting specificity. *I*, distribution of ^111^In-DTPA-11C12 and ^111^In-DTPA-control IgG in the brains of rats bearing tumors. *J*, distribution of ^111^In-DTPA-11C12 and ^111^In-DTPA-control IgG in the kidneys of rats bearing tumors. *K*, distribution of ^111^In-DTPA-11C12 and ^111^In-DTPA-control IgG in the liver of rats bearing tumors. *L*, tumor-focused images of mice inoculated with ^111^In-DTPA-11C12 and ^111^In-DTPA-control IgG. Maximum and minimum values of ID/g (injected dose per gram of tissue) are indicated by the *color bar*, where *white* represents the highest value, and *dark blue* represents the lowest value. *M*, longitudinal radial profiles of the distribution of ^111^In-DTPA-11C12 and ^111^In-DTPA-control IgG within tumors. Radial profiles of mean uptake were calculated for each tumor at each time point. The surface plots illustrate the tumor activity profiles as a function of time for 11C12 (*red*) and control IgG (*blue*).

To initiate the functional characterization of 11C12, we exposed human lung cancer cells (H460, H522, and A549) to increasing doses of IR in the presence or absence of 11C12 or a control isotype IgG and assayed for clonogenicity 10 days after irradiation (Fig. 7, *A–D*). Survival of A549 cells (low EphA5 expression) was not affected by 11C12 (Fig. 7*A*). In contrast, there was a marked reduction in the surviving fractions of H460 (Fig. 7*B*) and H522 (Fig. 7*C*) cells treated with 11C12, but not with the control IgG. Representative colony counts (H460 cells) illustrate this observation (Fig. 7*D*). Treatment of cells with 11C12 without radiation showed no significant changes in cell survival (data not shown).

Encouraged by these data, we used molecular imaging to study the accessibility of EphA5 *in vivo* and the biodistribution of radiolabeled 11C12. Because 11C12 cross-reacts with rat EphA5, but not with mouse EphA5 (Fig. 6*A*), these studies were performed in a rat xenograft model of human lung cancer (H460 cells). Rats were injected intravenously with ^111^In-DTPA-11C12 or ^111^In-DTPA-IgG, and single-photon emission computed tomography (SPECT) and CT images were acquired at 3, 24, 48, 96, and 144 h postinjection. Regions of interest corresponding to various control organs and tumors were defined for each subject in coregistered SPECT/CT images and were used as the basis for numerical analysis. Tumor-bearing rats receiving radiolabeled irrelevant IgG served as controls for targeting specificity. A second set of controls included non-tumor-bearing rats inoculated with radiolabeled 11C12 (data not shown).

Tumor targeting is clearly observed in animals receiving 11C12 (Fig. 7*E*), with the highest absolute uptake observed at 48, 96, and 144 h postinjection (Fig. 7*G*), compared with the control IgG (Fig. 7, *F* and *G*). In addition, the tumor to heart ratio of circulating 11C12 gradually increased over time, confirming antigen-mediated uptake within the tumor (Fig. 7*H*). Given that EphA5 is expressed in the developing and adult central nervous system, we also quantified 11C12 in the brain of tumor-bearing rats and found it to be at background levels (Fig. 7*I*). These results indicate that 11C12 is incapable of crossing the intact blood-brain barrier and reinforce its utilization as a therapeutic agent. Other control organs, such as kidney (Fig. 7*J*) and liver (Fig. 7*K*), showed nonspecific accumulation caused by clearance and excretion of the tracer. Importantly, tumor-focused images (Fig. 7*L*) and longitudinal radial profiles (Fig. 7*M*) of tumors from animals inoculated with 11C12 or control IgG revealed a highly homogeneous distribution of 11C12 within the tumor.

**FIGURE 8.**
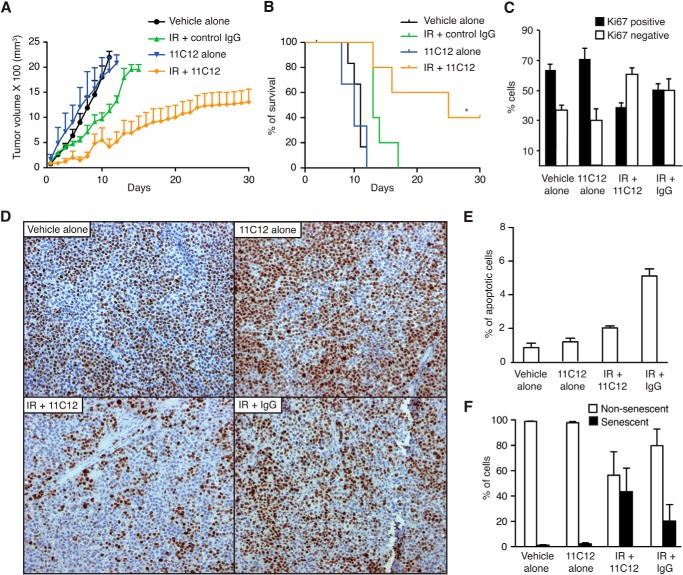
**The monoclonal antibody 11C12 radiosensitizes lung cancer *in vivo*.** *A*, growth inhibitory effect of the combined treatment of mAb 11C12 plus radiotherapy *in vivo*. Cohorts consisted of at least eight mice; the data are reported as mean tumor volumes ± S.D. *B*, Kaplan-Meier survival analysis. Combined treatment of 11C12 plus radiation therapy significantly improved the overall survival of tumor-bearing mice. *, *p* = 0.0061 (log rank test). *C* and *D*, quantification of Ki67-positive cells in tumor specimens collected 48 h after the end of each treatment. Photomicrographs illustrate Ki67-positive tumor cells. *E*, percentage of apoptotic cells in tumor specimens. Apoptosis was assessed by TUNEL staining. *F*, senescent cells in tumor tissue sections. Senescence was measured by positive staining with the SA-β-galactosidase method.

Next, we tested *in vivo* the therapeutic activity of 11C12 in combination with radiotherapy. Because of technical limitations associated with the use of rats for radiotherapy studies, we elected to perform these studies in mice bearing lung cancer xenografts (H460 cells). The targeting capacity and distribution of 11C12 in this animal model was evaluated and found to be comparable with that observed in rats (data not shown). The therapeutic effect of 11C12 was tested on four cohorts of mice (*n* = 8 each) bearing size-matched subcutaneous tumor xenografts and randomized into the following treatment groups: vehicle alone, IR plus control IgG, 11C12 alone, and IR plus 11C12. Fractionated radiation treatment consisted of 3 Gy of γ radiation per day for 3 consecutive days. As expected, tumor growth was delayed upon IR treatment in combination with control IgG. However, the combined treatment of 11C12 plus radiation therapy showed further, and more striking, inhibition of tumor growth (Fig. 8*A*). There was a highly significant increase in survival (*p* = 0.0061) (Fig. 8*B*), with none of the control mice surviving past 18 days, whereas 50% of the 11C12 plus IR-treated mice were alive 30 days later, at the end point of the study.

To evaluate the mechanism of action of 11C12 *in vivo*, we analyzed cell proliferation, apoptosis, and senescence in tumor specimens obtained from the different treatment conditions. A proliferation index, indicated by the percentage of Ki67-positive cells in tumor samples, was used to assess the effect of 11C12 on the proliferation of tumor cells. Animals treated with IR in combination with IgG control exhibited a ∼15% decrease in proliferation index compared with animals treated with vehicle alone or 11C12 alone. In contrast, the combined treatment of 11C12 plus IR resulted in a 30% decrease in the number of proliferating cells, a result suggestive of an anti-proliferation effect of 11C12 (Fig. 8, *C* and *D*). Interestingly, quantification of apoptotic cells in tumor samples showed low rates of apoptosis in all treatment groups (from 1% to 5%) (Fig. 8*E*), whereas quantification of senescent cells (as indicated by SA-β-gal activity in tissue sections) revealed a 40-fold increase in the number of senescent cells in tumors treated with a combination of 11C12 plus radiotherapy (Fig. 8*F*).

In conclusion, these experiments show that 11C12 increases the sensitivity of human lung cancer cells to radiation therapy and indicate that a combination of anti-proliferative and pro-senescence activities contributes to its therapeutic effects. These findings also support the view that monoclonal antibodies against EphA5 could have value in translational applications.

## DISCUSSION

In this study, we introduce the tyrosine kinase receptor EphA5 as a key regulator of radiation resistance in lung cancer through its novel and direct role in DDR. Several lines of evidence support this thesis. First, similar to ATM-deficient cells from ataxia telangiectasia (A-T) patients (34, 35), silencing of EphA5 in lung cancer cells *in vitro* caused severe defects in IR-induced G_1_-S cell cycle checkpoint and increased sensitivity to IR, suggesting that EphA5 may be participating in DDR as a component with ATM, well known to be a central kinase in DDR function(s) (36). Second, although we first recognized EphA5 as a cell surface receptor through phage display screenings on human cancer cells, we showed that it is also a cytoplasmic constituent that, like most transcription factors, translocates into the nucleus after irradiation and binds to chromatin. In fact, several membrane-bound receptor tyrosine kinases (RTKs), such as the EGF receptor and ErbB-2, have been reported in the nucleus of cancer cells (26–28). Recent evidence suggests that, in this subcellular location, these receptors are involved in essential cellular processes such as transcriptional regulation, cell proliferation, and chemo- or radioresistance (29–32). Furthermore, RTKs have been implicated in DNA repair processes (26, 27). For instance, EGF receptor has been shown to interact directly with DNA-dependent protein kinase (DNA-PK) and to regulate its activation upon exposure to genotoxic stress (27). Finally, we also showed a direct interaction between EphA5 and phosphorylated ATM at sites of DNA repair. Intriguingly, variations in *EPHA5* transcripts, and mutations in the cytoplasmic domain of EphA5 have been reported (37), raising the question of whether genetic and epigenetic factors may also play a role in EphA5 regulatory functions.

Whereas the molecular mechanisms by which EphA5 exerts its effects in the DDR remain to be fully determined, a few points merit further consideration. DDR activities are localized in the nucleus and are composed of multiprotein complexes (38, 39). The fact that EphA5 actively moves into the nucleus following DNA damage, as well as its interaction with phosphorylated ATM, indicates that EphA5 could be a member of such regulatory complexes. EphA5 does not have any of the classical amino acid nuclear localization signals, suggesting that it is transported into the nucleus in response to DNA damage as part of an activated protein complex (40). Among potential functions of EphA5 in the nucleus are stabilization of DDR complexes, facilitation of enzymatic processes, and possibly additional functions integral to DNA repair and checkpoint execution.

Following DNA injury, cells initiate a complex series of responses, including the activation of mechanisms of cell death and senescence. To maintain genomic stability, these pathways must be carefully balanced and coordinated. Given the functional description of EphA5 in both cell cycle checkpoints and DNA damage response, EphA5 is well positioned to serve as a mediator between these two pathways. Indeed, we showed that newly identified molecular cross-talk between EphA5-mediated pathways and p53 controls the terminal fate of cells exposed to IR: in the absence of EphA5, p53-proficient lung cancer cells become senescent, and in the absence of both EphA5 and p53, lung cancer cells die. Given the importance of cell death and senescence for the maintenance of genomic integrity, it is possible that EphA5 may be a central player in senescence responses such as replicative senescence and oncogene-induced senescence.

From a translational perspective, these findings have immediate clinical implications. Patients with locally advanced or distant metastatic adenocarcinoma or squamous cell carcinoma of the lung are generally not eligible for surgical resection of the tumor but must rely on radiotherapy, chemotherapy, and/or combined therapy modalities as the current standard of care (4, 5). Such aggressive therapy regimens not withstanding, acquired resistance to treatment and subsequent relapses are common and are followed by a high rate of tumor recurrence and death. Our immunohistochemical analysis showed that EphA5 is widely expressed in human lung cancer, particularly in squamous cell carcinoma and that those patients expressing EphA5 as a molecular marker are at much higher risk of radiotherapy failure and death. That ∼70% of lung cancer specimens analyzed in this study expressed EphA5 and that EphA5 expression in normal tissues is largely restricted to the developing central nervous system render EphA5 a suitable target for the development of systemic treatments, such as a monoclonal antibody-based targeted therapy. Indeed, our new monoclonal antibody against EphA5 (11C12), when administered in combination with radiotherapy, sensitized lung cancer cells to IR *in vitro* and improved the overall survival of mice bearing human lung cancer xenografts. Whereas anti-proliferative and pro-senescence mechanisms are implicated in the therapeutic effect of 11C12 in lung cancer, further preclinical experiments will be required to determine fully the mechanisms of action of the mAb 11C12 *in vivo*.

In conclusion, the therapeutic applications of EphA5-targeted therapy, together with its functions in the cellular response to DNA damage, identify EphA5 as a critical new target for therapeutic intervention in lung cancer. In fact, we have initiated a large scale effort to select new and improved anti-EphA5 antibodies through a hierarchical approach that combines phage and yeast display (33). This selected panel of human antibodies will possess specific biological properties of therapeutic interest and will support the introduction of EphA5-targeted therapies into the clinic. Likewise, we have also detected EphA5 in tumors other than lung (data not shown), raising the possibility that EphA5 might be a molecular target in other human cancers.

## Footnotes

6 The abbreviations used are:

IR: ionizing radiation
DDR: DNA damage response
DTPA: diethylene triamine pentaacetic acid
TMA: tissue microarray
SA: senescence-associated
ACC: adenocarcinoma
SCC: squamous cell carcinoma
pATM: phosphorylated ATM
SPECT: single-photon emission computed tomography
CT: computed tomography
RTK: receptor tyrosine kinase.
